# Cell Surface and Membrane Engineering: Emerging Technologies and Applications

**DOI:** 10.3390/jfb6020454

**Published:** 2015-06-18

**Authors:** Christopher T. Saeui, Mohit P. Mathew, Lingshui Liu, Esteban Urias, Kevin J. Yarema

**Affiliations:** 1 Translational Tissue Engineering Center (TTEC), Johns Hopkins University, 400 N. Broadway, Baltimore, MD 21287, USA; E-Mails: chris.saeui@gmail.com (C.T.S.); m.mohit0930@gmail.com (M.P.M.); lliu48@jhu.edu (L.L.); eurias99@gmail.com (E.U.); 2 Department of Biomedical Engineering, Johns Hopkins University, Baltimore, MD 21231, USA

**Keywords:** cell surface engineering, membrane engineering, metabolic engineering, biofuel synthesis, biosensors, bio 3D printing

## Abstract

Membranes constitute the interface between the basic unit of life—a single cell—and the outside environment and thus in many ways comprise the ultimate “functional biomaterial”. To perform the many and often conflicting functions required in this role, for example to partition intracellular contents from the outside environment while maintaining rapid intake of nutrients and efflux of waste products, biological membranes have evolved tremendous complexity and versatility. This article describes how membranes, mainly in the context of living cells, are increasingly being manipulated for practical purposes with drug discovery, biofuels, and biosensors providing specific, illustrative examples. Attention is also given to biology-inspired, but completely synthetic, membrane-based technologies that are being enabled by emerging methods such as bio-3D printers. The diverse set of applications covered in this article are intended to illustrate how these versatile technologies—as they rapidly mature—hold tremendous promise to benefit human health in numerous ways ranging from the development of new medicines to sensitive and cost-effective environmental monitoring for pathogens and pollutants to replacing hydrocarbon-based fossil fuels.

## 1. Introduction

The interface between the cell membrane and the extracellular matrix is a critical barrier that signaling molecules, other cells, pathogens, and environmental cues must traverse to modify cellular physiology. The membrane bilayer, however, is not just an obstruction but has evolved to be an exquisite enabling mechanism for signal transduction that contains a myriad of inserted transmembrane and carrier proteins that act as channels, transporters and signal transduction mediators that serve critical functional roles in adhesion, cellular communication, and bridging the extracellular environment with the cellular cytoskeleton. These factors position membranes as prime engineering targets to develop platform technologies ranging from whole cells to synthetic constructs based on concepts derived from cells. Applications for these technologies range from research tools that facilitate drug discovery and help understand complex cell-cell interactions to the creation of non-natural transmembrane protein systems for purposes including energy production, biosensing technology, and mitigation of environmental pollutants. Many *in vitro* diagnostic assays and screening tools already rely on technology that includes engineered membrane systems, thus there is not only a pressing need for engineering strategies to further advance basic research through the manipulation of the cellular membrane and its components, there is also a substantial demand for the translation of these technologies into the commercial marketplace. For example, according to one recent market analysis [1], the global market for life science tools and reagents reached $51.3 billion in 2013 and is expected to grow to $77.6 billion in 2018.

In this article, we outline emerging strategies for engineering the cellular membrane by first providing an overview of technologies that utilize living cells and then by introducing cell-free systems inspired by nature (in Section 2). Next, in Section 3, Section 4, and Section 5 we provide illustrative examples of membrane-based and inspired technologies that focus on drug development and testing, biofuels, and biosensors, respectively. These examples include a mix of applications that use living cells and a sampling of their extension to entirely synthetic systems to provide perspective on the advantages and pitfalls of each approach. Finally, in Section 6 we explore the potential contributions of bio-3D printing technology, which although still in its infancy, promises to be a powerful enabling tool in recapitulating cell membrane biology in an *in vitro* setting.

## 2. Basic Strategies: Cell-Based *vs.* Synthetic Systems

Cell-based membrane platforms are actively being developed for purposes ranging from biosensing, biofuel synthesis, and drug screening and technologies used to achieve these goals include installing a protein or system of proteins into the lipid bilayers of a cell through genetic manipulation, microinsertion, or by engineering the biogenesis of membranes by including lipid production as a design parameter. Although cell-based platforms offer important advantages such as fast response times in a physiologically relevant setting and naturally contain much of the machinery needed for recombinant protein synthesis they also have drawbacks. For example, they often suffer from a limited shelf life, a strict requirement for aseptic techniques, and the time investment needed to grow cells. In order to address these issues, cell-free synthetic systems pursue many of the same goals while avoiding the complications arising for the complexity of living systems. Accordingly, to provide perspective on both approaches, in this article we highlight a sampling of emerging technologies to illustrate both cell-based and cell-free platforms.

### 2.1. Biological Membrane Systems

Living cells encompass an elegant, sophisticated, and versatile platform for membrane-based technologies and attempts are underway to utilize them for applications ranging from drug development, protein over-expression, biosensing, to pollution mitigation. These applications typically require the biomolecular engineering of transmembrane proteins, which remains difficult to achieve in many cases and to further complicate matters, a lipid bilayer must be embedded with proteins that are properly folded and correctly oriented to create a fully functional biological membrane. In mammalian cells, membrane proteins depend on an extraordinarily complex quality control system during manufacture that has at its core the molecular chaperone proteins calnexin and calreticulin that ensure correct folding; this system also critically depends on N-glycosylation [2,3]. Because N-glycosylation relies on proteins encoded by about 3% of the genome in humans, its hundreds of constitutive components make it virtually impossible to duplicate in a cell-free system. This quick example illustrates the value of cell-based systems that have evolved exquisite biosynthetic machinery for properly folding water-insoluble proteins and concomitantly inserting them into membranes in a single and controllable orientation.

In some cases, increased expression of transmembrane proteins already present in a cell is the desired membrane engineering endpoint but more commonly the design goal is to insert proteins that are not normally found in host cell. For example, most systems designed to be biosensors or for the mitigation of environmental pollutants require the introduction of membrane proteins—or in some cases an entire system of transmembrane proteins—not normally found in the host cell. In either case, there is a requirement to not only over-produce properly folded and biologically active proteins but to also manipulate the cellular machinery responsible for membrane biosynthesis because insufficient lipid bilayer biogenesis can profoundly limit the amount of protein ultimately produced. Another problem that often arises is that particular classes of proteins including membrane transporters, porins, and channel-forming proteins that control membrane permeability and affect delicate intracellular chemistry tend to be toxic to host cells [4]. Consequently, engineered membranes designed to implement these classes of proteins often demand alternative production strategies, including cell-free, synthetic membrane systems.

### 2.2. Cell-Free, Artificial Membrane Systems

The engineering of membrane biology in cell-based systems is yielding insights that are aiding in the design of manufactured platforms that strive to mimic cellular biology through entirely synthetic “artificial membrane” systems. Advantages of synthetic platforms include the potential for higher throughput, greater stability from degradation for both reagents and products, and simplified experimental systems that result from avoiding competing reactions already present in a cell’s metabolic networks. Examples of promising advances resulting from early stage implementation of cell-free synthetic platforms include MembraneMax™ protein expression kits that utilize nanodisc biolayer and Arrowhead Research Corporation’s successful phase I clinical trial of CALAA-01, a polymersome that delivers patented siRNA against the M2 subunit of ribonucleotide reductase (RRM2) by inserting transferrin into the polymersome to target transferring receptors that are often over-expressed in cancer [5]. One disadvantage of cell-free systems, as already mentioned, is the fundamental difficulty of obtaining properly folded, biologically active membrane proteins without the elaborate quality control systems found in cells. Encouraging, even vexing problems such as this one are being overcome through exciting technologies that promises to overcome manufacturing challenges such as bio-3D printing, as discussed below in Section 6 of this chapter.

### 2.3. Characterization Technologies are Propelling Membrane Engineering Applications

Based on the promise of synthetic, cell-free membranes, there have been recent attempts to fabricate artificial systems using nanostructures that mimic the physiological properties of natural lipid bilayer membranes fused with transmembrane proteins. As discussed in a comprehensive review [6], temperature, pH, ionic strength, adsorption behavior, conformational reorientation and surface density are all important factors that affect the incorporation of proteins into membranes. Clearly, to be able to assess the myriad of possible multifactorial conditions, methods and technologies must be available to characterize artificial membranes in a rapid, inexpensive, and user-friendly manner and—ideally—directly compare them with biological membranes. Very briefly, one such method that is gaining credence for verifying peptide and protein insertion into both natural and synthetic membranes is electrochemical impedance spectroscopy (EIS) [6,7,8]. Additional methods for charactering membrane composition include quartz crystal microbalance (QCM) [7], surface plasmon resonance (SPR) spectroscopy [7,9] as well as “nanoscale” methods such as light scattering spectroscopy (LSS) and surface enhanced Raman spectroscopy (SERS) [9]. While in no means meant to be comprehensive, this list is meant to illustrate that several complementary and powerful techniques are becoming increasingly available and affordable and promise to propel forward the technologies described in this article.

## 3. Biomedical and Pharmaceutical Applications

Membrane-based technologies are critical in biomedicine—in particular in drug development and production—in several ways. An overview of this topic is given here in Section 3, beginning with a brief discussion of the biomedical relevance of membrane proteins (Section 3.1). Next, a cell-based solution to overcome difficulties in achieving robust levels of membranes proteins often required for bioassays used in drug testing, which is concomitant overproduction of lipid components of membranes that in turn facilitates membrane protein production is covered in Section 3.2). Finally, the development of cell-free synthetic systems for the display, study, and utilization of membrane proteins for biomedical applications and drug development is covered in Section 3.3.

### 3.1. Membrane-Based Receptors are Critical, but Difficult to Study, Drug Targets

Early stages of drug development, an integral aspect of the pharmaceutical industry, currently has a poor record of predicting therapeutic candidates that will ultimate experience clinical success; for example phase II testing eliminated ~82% of compounds as viable drug candidates [10]. Clearly, accurate and improved *in vitro* tools in preclinical work are desperately needed to identify and guide early stage drug candidates through development with improved odds of translatable success into the clinic. Because of abysmal failure rates of lead candidates during clinical testing reasonably reflects back to insufficient models for testing drugs at early stages of development, much effort is devoted to elucidating the mechanisms by which novel drug molecules act, understanding the biochemical pathways they impact, and the cell wide molecular interactions they make because these factors are crucial for predicting efficacy and the potential for adverse effects for new drug candidates [11]. The relative lack of success in these efforts, however, can be traced to observations that medicines ubiquitously target membrane proteins and the fact that membrane proteins remain remarkably difficult to study using even the most sophisticated tools of modern molecular and cellular biology.

To provide perspective on the magnitude of this challenge, collectively integral membrane proteins constitute approximately 30% of any given proteome [12], with a subset of these proteins, G-protein coupled receptors (GPCRs) comprising 4% of the entire protein encoding genome [13]. GPCRs encompass a family of receptor proteins with a seven member transmembrane domain and are the targets of 50% of current drugs. This remarkable family of proteins has hundreds of members and their significant diversity greatly complicates the drug discovery process. Compounding the problem, GPRCs are membrane-bound proteins bringing a whole new set of challenges into play. To illustrate one relevant challenge, Nobel Prize winner Max Perutz first solved the crystal structure of a protein (hemoglobin) in 1959 and numerous protein crystal structures have been obtained over the past half century. Obtaining the crystal structures of cell surface protein receptors still remains a formidable challenge, however, with less than 20 GPCR crystal structures have been solved (GPCRSD database http://zhanglab.ccmb.med.umich.edu/GPCRSD/) despite their incredible importance as drug targets. In general, even today obtaining detailed biochemical information through structural characterization of membrane bound proteins ranges from being “quite tedious” to being completely intractable due to the technical difficulties associated with obtaining sufficient quantities of properly folded membrane proteins for crystal structure determination. Isolation of membrane proteins involves extraction with detergents, refolding with detergent (which is particularly difficult in the absence of *N*-glycan assisted chaperone protein processes [2]), and in some cases, reconstitution with liposomes; all of these steps invariably can lead to difficulties in obtaining the biologically-relevant forms of proteins that are desired during structural analysis.

### 3.2. Overcoming Challenges in the Over-Expression of Membrane Proteins

Despite numerous advances in molecular biology technologies over the past few decades, the overexpression and structural characterization of membrane proteins remains challenging, thereby hindering the tremendous potential of these methods to guide drug discovery. An example of such a benefit lies in estimates that structure-based drug design (SBDD) can reduce costs of drug discovery from target identification to investigational new drug (IND) filing by almost 50% largely due to the improved quality of lead candidates that results from high quality structural information [14,15]. One example of successful SBDD against a cell surface receptor has been the development of agonists against neuronal nicotinic receptors for the treatment of addictions, Alzheimer’s related dementia, and glaucoma. Technology that streamlines SBDD through simplification of the X-ray crystallography process could tremendously improve the success rate and time needed for development of new small molecule-based drugs.

#### 3.2.1. Cells Have a Limited Capacity to Produce Membrane Components, Including Embedded Proteins

One of the first bottlenecks that must be overcome to tackle a structure-function protein biology problem—for example, to determine how a drug candidate interacts with a GPCRs—is obtaining the hundreds-of-milligram quantities of protein required for X-ray crystallography. While bacterial (e.g., *Escherichia coli*) expression systems have long been used to generate water soluble, usually cytosolic, recombinant proteins [16], membrane proteins are more challenging because they contain critical features such as phosphorylation and glycosylation that *E. coli* cannot accommodate without further engineering. Post-translational modifications such as phosphorylation are widely recognized to control protein function and require no further discussion here. The role of glycosylation is often not fully appreciated but cannot be overlooked because it is critical to properly fold a protein for compatibility with the lipophilic environment of the cell membrane [2,17,18]. Therefore eukaryotic systems, such as yeast, capable of producing post-translational modifications, in particular glycosylation patterns that are roughly similar to those found in mammals [19], are being considered as host organisms to overexpress membrane proteins [20]. Insect cells that utilize baculovirus protein expression [21,22] are particularly enticing insofar as they not only provide sufficient amounts of high quality protein for crystallization but also include efforts to produce favorable glycosylation patterns [23]. Even one of the most vexing issues with insect expression, the lack of “human”-like sialylated glycans [24,25], is beginning to yield to indications that insect cells do have the ability to produce sialic acid and incorporate this unusual sugar into their glycoproteins [26,27].

Even with the availability of a eukaryotic systems such as yeast, insect cells, or increasingly Chinese hamster ovary (CHO) cells [28,29], production of membrane proteins in sufficient quantity to produce high quality crystals for structure determination often remains daunting. Very few membrane proteins have sufficiently robust expression from their natural cellular milieu to allow the high levels of purification required for crystallography. The challenge in the “obvious” solution—the over-expression of recombinant membrane proteins, often induces toxicity, protein aggregation, and misfolding that result in overall low yields of properly folded and biologically relevant products that continue to stymie X-ray crystallography studies [30]. The foundation of this problem is apparent when considering the canonical “fluid mosaic” model of the plasma membrane where lipids and proteins are each present at ~50% abundance by mass. Based on the high protein content, an argument can be made that a typical “lipid” membrane is already at capacity with respect to protein content and it is unsurprising that attempts to overload a membrane with a recombinantly-expressed protein often encounter difficulty. Consequently, efforts to express membrane proteins should consider not only the steps needed to express the protein but also take measures to commensurately increase lipid production to meet the need for increased membrane surface to accommodate these proteins. While the discussion in this section is directed towards obtaining sufficient material for X-ray crystallography, a similar approach is expected to be valuable in the biotechnology industry for recombinant glycoprotein production or for the orientation of polysaccharide-processing enzymes in the outer membrane of microorganisms for biofuels production, as discussed later (in Section 4) in this article. The next section will highlight recent engineering efforts that seek to overcome the limits of currently employed *in vitro* systems used to study cell membrane proteins with a focus on strategies to manipulate cellular membrane synthesis as well as recent ideas designed to isolate larger quantities of membrane proteins using cell-free systems.

#### 3.2.2. Manipulating Lipid Metabolism

Efforts are underway to understand membrane biogenesis in more depth to learn how to manipulate the eukaryotic endomembrane synthetic machinery to increase surface area so as to accommodate accumulating integral membrane protein levels in engineered expression systems [31,32,33,34]. This approach, while in its infancy, is a promising strategy for the isolation of sufficient quantities of integral membrane proteins for applications such as high quality X-ray crystallography. Unlike protein synthesis however, which is driven by the DNA code, no simple template exists that can control the metabolism of lipids and direct the synthesis of membranes. Consequently, a thorough understanding of the biochemical pathways that lead from lipid to membrane, with an identification of key bottleneck enzymes, is required. Furthermore, nutrient supplementation and manipulation of the extracellular environment—often in conjunction with genetically engineering lipid metabolism—are needed to control metabolic flux through lipid metabolic pathways in order to divert intermediate lipid metabolites towards incorporation into lipid bilayers.

**Figure 1:**
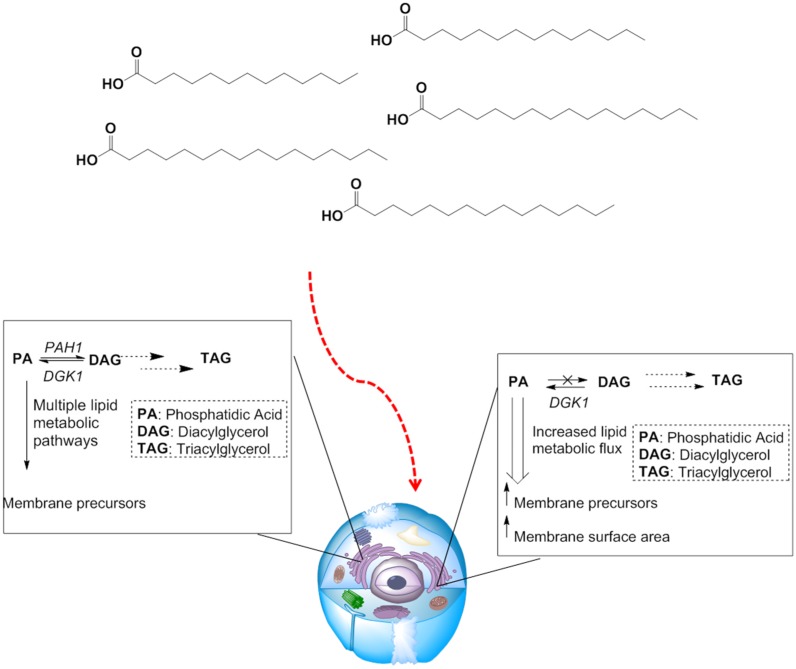
Redirecting lipid metabolism to membrane biogenesis. When culture systems are supplemented with fatty acids, large portions are stored as DAG and TAG (left). Increased lipid metabolic flux can be diverted towards membrane biogenesis by deleting the *PAH1* gene (right), inhibiting storage of fatty acids. Increased lipid flux towards membrane precursor biosynthesis leads to a larger total membrane surface area, provided a cell with an increased membrane capacity leading to higher yields for over-expressed membrane proteins.

Fatty acids are absorbed from culture media by eukaryotic cells and then stored in lipid droplets in the cytoplasm as triacylglycerols (TAG) and steryl esters (SE) [35]. If storage as TAG and SE is suppressed, flux of fatty acids and their metabolites into anabolic pathways and towards new membrane construction is promoted. In particular, *PAH1*, the gene that encodes phosphatidic acid phosphatase (Pah1p), acts as a gate keeper for partitioning flux of fatty acids into storage as TAG [36]. This gene has become a target for genetic knockdown in yeast because if *PAH1* is deleted, diacylglycerol (DAG), and subsequently TAG, biosynthesis is disabled and metabolic flux in a cell is redistributed to produce excess membranes (Figure 1) that facilitate the overproduction of biologically active integral membrane proteins by providing an increased membrane surface area [37].

The regulation of genes involve in fatty acid metabolism is well established making it possible to engineer *Yarrowia lipolytica*—a ‘‘non-conventional’’ yeast capable of producing important metabolites and having an intense secretory activity, for use in industry as a biocatalyst, in molecular biology, and in genetics studies [38] for increased membrane production by creating strains with *PAH1* deleted genes (Δ*pah1*) [37]. Growth studies using either glucose or oleic acid as a carbon source revealed that *Y. lipolytica* wild type and Δ*pah1* strains had similar proliferative capacities, indicating that deletion of *PAH1* has no serious impact on viability that would preclude the knockout strains from being used as hosts for producing recombinant membrane proteins. Next, the adenosine A_2A_ receptor (A_2A_R), a member of the GPCR superfamily, was expressed by integrating the A_2A_R gene under the oleic acid-inducible POX2 promoter in both wild type and Δ*pah1* strains of *Y. lipolytica*. Furthermore, to increase the capacity of *Y. lipolytica* for protein production, the endoplasmic reticulum quality control mechanism for unfolded proteins, also known as the unfolded protein response (UPR), was concomitantly engineered by transforming both the wild type and Δ*pah1* strains with spliced active *HAC1*, a non-canonical intron mRNA that leads to translation of transcription factor Hac1p that activates the UPR, which was also driven by the POX2 promoter. Hac1p is found only in ER stressed yeast that has accumulating unfolded protein in its endoplasmic reticulum [39], thus the motivation for creating Δ*pah1* strains was to fully integrate strains of yeast with increased membrane biogenesis with other metabolic engineering targets that have been identified as critical control mechanisms to regulate the synthesis of properly folded proteins.

Western blot analysis showed that the Δ*pah1* mutation profoundly enhanced production of over-expressed recombinant transmembrane protein A_2A_R [37]. Furthermore, quantification of mRNA levels revealed that enhanced A_2A_R production was due to not stronger promoter activation; rather, the driving force that led to higher quantities of A_2A_R was the deletion of *PAH1* and the simultaneous over-expression of *HAC1*; the Δ*pah1/Hac1p* combination resulted in the production of large quantities of fully functional A_2A_R through enhanced ER quality control coupled with increased membrane synthesis. Increased membrane synthesis was rigorously documented by electron microscopy, which showed that the Δ*pah1* mutant strains produced an increased amount of intracellular membranes compared to that wild type strain. The wild type strain produced massive quantities of TAG that were observed as intracellular lipid droplets when the yeast was grown on oleic acid. In contrast, the Δ*pah1* mutant strains were almost completely devoid of droplets in agreement with the hypothesis that enhanced lipid flux within the Δ*pah1* mutated strains was diverted towards membrane biogenesis. Finally, as a demonstration that these membrane engineering techniques were broadly applicable, the production of additional membrane proteins (human 5HT_1D_ receptor, human mu opioid receptor, aquaporin Aqy1 of *Pichia pastoris*, human B-cell associated receptor 31, porcine respiratory virus (PRV) NS4, presenilin/SPP homolog of *Methanoculleus marisnigri*, and human cytochrome P450 2D6) were evaluated in Δ*pah1* mutant strains and all showed enhanced expression [37].

### 3.3. Synthetic Membrane-Based Systems for Drug Development

As discussed above, there are many complications and experimental challenges associated with solving crystal structures for GPCRs and other cell surface proteins. However, overcoming one of the first bottlenecks—the task of simply obtaining a sufficient quantity of high quality cell surface protein—may soon be solvable through cleverly designed cell membrane engineering strategies that increase membrane surface area sizes coupled to endogenous protein folding correction machinery as discussed above. Once a facile production system is in hand, additional opportunities arise for the study and exploitation of GPCRs and membrane proteins in general. For example as described here in Section 3.3, GPCRs can be embedded in synthetic membranes and thus serve as emerging *in vitro* tools and reagents to aid drug screening and capture of transmembrane proteins. The need for these technologies is illustrated by GPCRs; biologically they gain their role as a prevalent drug because they initiate signaling cascades [40] implicated in diseases ranging from those that impact the nervous system such as Alzheimer’s and Parkinson’s diseases to seemingly unrelated diseases such as Nephrogenic diabetes insipidus and other disorders of the cardiovascular systems and metabolic syndromes [41]. While effective, GPCR-acting drugs still face the common problem of achieving stringent specificity for one GPCR over another to avoid deleterious off-target effects. As described earlier, one way to address this problem is through the use of membrane engineering in living cells to obtain high quality protein for X-ray crystallography. Another direction being exploited to obtain specificity is through the use of proteopolymersomes [42], a specialized type of polymersomes that holds promise as a valuable *in vitro* testing platform that enables (amongst other capabilities) screening of mutations associated with disease.

#### 3.3.1. Polymersomes for Drug Development

Polymersomes are cell-sized artificial vesicles typically having a diameter of 100 nm to 10 μm that usually contain an aqueous solution in their core laden with biological molecules such as drugs, enzymes, or nucleic acids. Polymersomes can be thought of as “second generation” liposomes that have overcome limitations such as fragility, sensitivity to oxidation, and instability due to the inherent labile nature of lipids and lipid constructs by incorporating synthetic block copolymers into vesicle design. Polymersomes are generally designed to be encapsulated containers for delivery of sensitive biological agents or to serve as internal reaction chambers cordoned off from the surroundings by the membrane. Increasingly, they also offer a promising avenue for the display and production of membrane proteins; to date, several examples exist where membrane proteins have been introduced into a polymersome bilayer [42,43,44]. Polymersome production is not without its own challenges however. Poor size control and low efficiency of encapsulating the machinery needed to generate membrane proteins remain significant roadblocks to the widespread adoption of this emerging technology.

**Figure 2:**
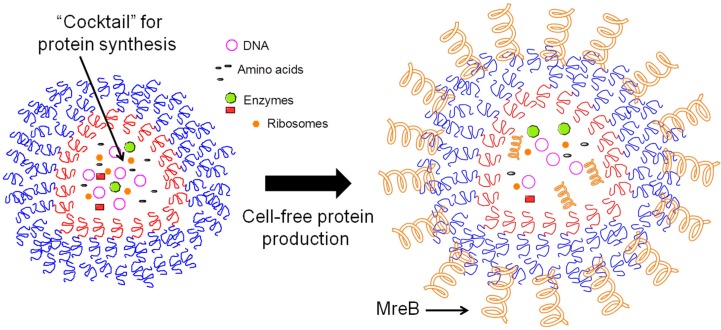
Production of self-assembled polymersomes. Self-assembled polymersomes can be created utilizing microfluidic techniques to introduce a cocktail containing the machinery required for protein synthesis (inner layer, left) through a microchannel into an oil layer-containing polymer (middle layer). The nascently–formed spheres flow through the oil layer into an aqueous phase, forming polymersomes with the outer blue PEG-PLA shown on the left. Cell-free protein production takes place in these constructs; for example, as cited in the main text, the bacterial protein MreB (orange spring) can be produced after which it spontaneously diffuses and inserts into the polymersome membrane.

A recent approach [44] for synthesizing membrane proteins in a polymersome utilizes microfluidic techniques to capture ribosomes, amino acids, DNA templates, and an enzyme mix to create a bioactive cocktail that contains the essential machinery that cells employ in protein synthesis. In the example illustrated in Figure 2, bacterial membrane protein MreB was expressed within a polymersome constructed of poly(ethylene glycol)-*block*-poly(lactic acid) (PEG-*b*-PLA); the PLA homopolymer was added to increase the hydrophobicity of the polymersome membrane. By using this strategy, a proportion of MreB synthesized within the polymersome migrated and anchored into the polymer bilayer despite the absence of the elegant secretory apparatus found in eukaryotic cells due to interaction between a hydrophobic helical domain within the MreB protein and hydrophobic polymer membrane. In a second example [40], polybutadiene-polyethyleneoxide (PBD-PEO) polymersomes were constructed and claudin-2 (Cldn2), a membrane protein important in cell-cell interactions, was used as a model for insertion into the polymer bilayer. In this case, Cldn2 was synthesized extrinsically using a standard wheat germ protein expression kit, a common technique for cell-free protein expression [45]. By including the polymersome in the reaction mixture during Cldn2 expression, surface plasmon resonance (SPR) confirmed that Cldn2 spontaneously inserted into the polymersome.

While this early proof-of-principle example provides evidence that polymersomes can serve as vehicles for display of membrane proteins as “proteopolymersomes,” several questions remain including whether the proteins that insert into the polymersome membrane are correctly folded (due to a lack of quality control mechanisms found in cells, as discussed early), biologically active, and consistently oriented inwards or outwards as desired. Additionally, several challenges remain for reproducible, robust, and scalable synthesis of polymersomes. Cell-free membrane expression systems that utilize a polymersome platform as cell-membrane mimetics hold promise as they can potentially circumvent the difficult problems that inevitably arise with overexpressing integral membrane proteins in cellular systems such as incorrect folding, cytotoxicity, and protein aggregation. Polymersomes can also avoid the labile nature of cell-free expression systems that use liposomes or other lipid based structures to create other artificial bilayer structures. Artificial bilayer structures and synthetic membranes are faster and a more economical alternative to animal or cell-based testing. Moreover, the use of synthetic membranes allows for large scale industrial testing capabilities, which accelerates the drug discovery process. Examples of institutions researching synthetic membranes include Agency for Science, Technology and Research’s (A*STAR) Artificial Cell Membranes (ACMs) Biolabs and the U.S. Department of Energy’s Argonne National Laboratory. Generating synthetic membranes with functional membrane proteins can thus create nearly identical, chemically controlled environments in which the activity of drugs can be tested. Synthesis of the artificial membranes involves mainly polymeric materials together with membrane proteins that are embedded in the nanosphere structure. Because target proteins are inserted into artificial membranes in isolation, prevention of additional background noise related to non-specific binding of drug molecules is achieved, resulting in lower interference signals in binding and transport assays.

#### 3.3.2. Nanolipoprotein Biolayer Discs for Membrane Proteins

One novel approach to present functional membrane proteins in a defined and easy-to-study manner has involved the development of nano-sized lipid bilayer discs referred to as nanodiscs or nanolipoprotein particles (NLPs) [46,47,48,49]. It is well known that phospholipids self-assemble into bilayers, with the most common form being a liposome. Although liposomes resemble cell membranes and are regarded as the gold standard model of a membrane when studying membrane-embedded proteins, liposomes are not without their drawbacks. For example, liposomes are often large, difficult-to-characterize aggregates that are microns in size and contain hundreds of millions of phospholipids. Detergents can be used to reduce liposome size to make constructs easier to study; these reagents, however may also denature essential proteins in the process [46]. NLPs offer an attractive alternative because they can be synthesized on the order of nanometers (with each NLP containing only about 150 phospholipids), offer access to both sides of NLP disc, and overcome the problem of correct protein orientation within a lipid bilayer.

**Figure 3:**
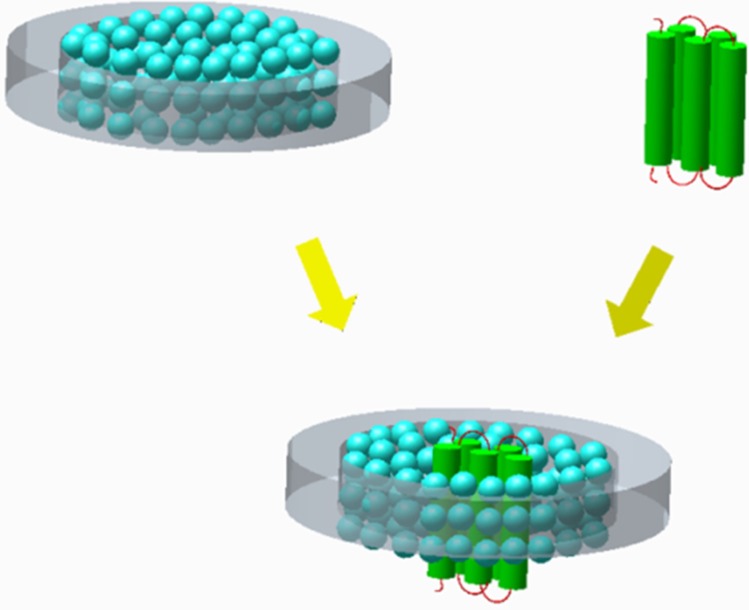
Protein insertion into nanolipoprotein particles (NLPs). Two amphipathic α-helical proteins (gray ring) self-assemble with lipids to produce a nano-sized disc. When introduced into a protein expression system, certain membrane proteins spontaneously insert into the NLPs.

Briefly, NLPs can be synthesized by utilizing amphipathic α-helical proteins that can self-assemble into bilayer disc-like structures with lipids, where the edge of the discs are defined by the α-helical proteins that stabilize the leaflets [50,51,52,53,54] (Figure 3). α-Helical proteins can initiate nanodisc formation using detergent dialysis [50] that, when coupled with the titration of a desired lipid at an optimized lipid to protein ratio [46], can spontaneously form homogenous ~10 nm-sized bilayer discs. When cell-free protein expression systems are used to generate a membrane protein of interest, protein-NLP complexes can form *in situ* [45]. Finally, radiolabel-based ligand assays can be performed after purification to verify by binding or catalytic activity whether the membrane protein properly folded and inserted into the NLPs. Recently, a diverse range of membrane proteins have been produced, incorporated into NLPs, and verified to be in a correctly folding conformation [55,56,57,58,59,60]. The increasing success and robustness of this technique may soon make the expression of membrane proteins in NLPs a common and routine laboratory technique that becomes standard for the general study and exploitation of membrane proteins. As a caveat, many proteins do not spontaneously insert into lipid bilayers and therefore are not immediately amenable for NLP incorporation; however as the basic requirements for protein insertion into membranes become unraveled [61,62,63] it is anticipated that peptide sequences can be rationally designed to widely facilitate NLP production for an increased repertoire of proteins.

## 4. Biofuels Production

**Figure 4:**
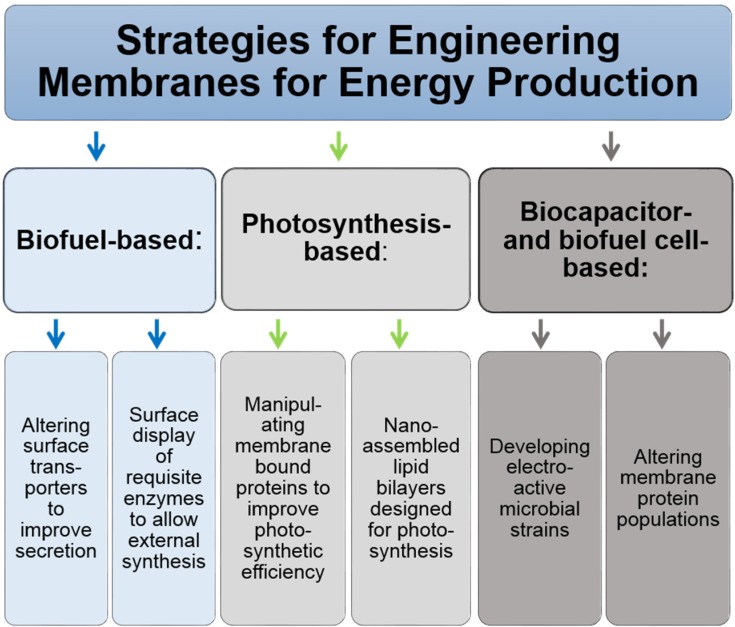
Overview of strategies for biofuel synthesis where membrane engineering has a central role.

Based on the vast amounts of solar energy available (as outlined below in Section 4.1), biofuel production is another active area of research where membrane engineering in living cells is fundamentally important. In the past, much of the research in this area has focused on altering the internal enzymatic machinery to optimize product formation; these efforts continue today as exemplified by the strategy described in Section 4.2 of minimizing wasteful “protective” mechanisms in host organisms used for biofuels production. It is becoming increasingly evident, that membrane surface engineering also can play a role in improving the efficiency of biofuel synthesis as well as recovery and purification (Figure 4). One strategy to improve biofuel production is to increase the export of intracellularly produced biofuel products out of the host cell, which involves engineering channels or transporters into surface membranes (Section 4.3). An alternative strategy involves moving biofuel production from the intracellular environment by installing enzymes capable of producing biofuels onto the surface of cells (Section 4.4), thereby bypassing the need for extracellular transport at all. Finally, the use of biomass to generate electricity directly is covered in Section 4.5 and emerging efforts to reproduce photosynthesis in cell-free systems is outlined in Section 4.6.

### 4.1. The Promise of Photosynthesis-Based Fuel Production

Incident solar radiation is theoretically capable of supplying 178,000 TWh of energy per year [64], which would be sufficient to meet the Earth’s current energy requirements of ~110,000 TWh per year (for example, ~100,000 TWh of energy was consumed in a recent year (2012) [65]). Certain challenges in meeting this objective, such as the diffuse nature of solar energy that renders it difficult to harvest and the high expense of solar energy capture technologies [66] are largely beyond the scope of this discussion. We will therefore focus on only one technology that is based on an age-old biological system—photosynthesis, which is the planet’s oldest, most prevalent and most efficient solar capture mechanism—to inspire cost-effective systems with enhanced photon capture efficiency [66]. Specifically, this discussion will be focused on the challenge posed in transforming solar radiation into chemicals capable of substituting for the ~80% of global energy usage currently met by hydrocarbon-based fossil fuels. One way to pursue this objective is to directly link the electrons and protons produced in the initial stages of photosynthesis with hydrogenases (enzymes that convert protons and electrons into hydrogen) to produce hydrogen as a biofuel [67]. In a slightly more convoluted manner, conversion of biomass produced by photosynthetic processes into liquid fuels is a tangible way to utilize solar energy by exploiting nature-inspired, membrane-based technologies. Membrane-based approaches can facilitate these technologies several ways that include increasing the efficiency of biomass production (Section 4.2), enhancing recovery of intracellularly-produced fuels (Section 4.3), and engineering surface assemblages of enzymes for biomass processing (Section 4.4).

### 4.2. Manipulating Existing Photosynthetic Pathways to Avoid Inefficient Steps

Photosynthesis has an initial light-to-charge conversion reaction efficiency of ~50%, which is more than double the output of current state-of-the-art commercial photovoltaic systems. Subsequent losses required for photoprotection and thermodynamic inefficiencies (necessary for complex networks of competing reactions), however, lead the overall energy conversion efficiency to be much lower [66,68]. As a result, multifaceted approaches seek to improve the efficiency of photosynthesis-based solar fuel production [67]. Pursuing this objective starts with efforts to engineer existing photosynthetic pathways to avoid inefficient steps. The “inefficient steps” in photosynthesis are now realized to be a consequence of evolutionary pressures to design robust systems that maximize the survival and reproduction of organisms.

A specific example of this paradigm is provided by the Light Harvesting Complex (LHC) proteins involved in photosynthesis. LHCs are thylakoid membrane-bound proteins that bind pigments such as chlorophyll and carotenoids and orient them in an optimal configuration for photon capture. LHC proteins are finely regulated from level of mRNA transcription to protein degradation [69,70,71,72,73,74,75] and not only serve to capture solar energy but also provide plants with a protective adaptive purpose. In particular, when the level of solar energy exceeds the energy demand of the cell, LHCs are down regulated in order to prevent photo-damage. Conversely, in low light, LHC transcription increases to maximize photon capture efficiency [76]. When irradiation intensity exceeds photosynthetic capacity, LHCs help to dissipate energy as heat or fluorescence, thus protecting a plant from oxidative damage [77]. This protective mechanism, however, dramatically decreases the overall energy conversion efficiency of the host organism because nearly 80%–90% of the energy from photons is lost as heat and fluorescence during times of over-irradiation [77,78,79,80]. In an industrial setting where solar intensities can be controlled and where efficiency is of the highest priority, modification of the cells used for production to dispense with protective—but energy wasting—LHC proteins is a sound strategy. An illustration of this concept is provided by mutants of the algae *Chlamydomonas reinhardtii* that were engineered with RNAi technology to permanently down-regulate LHC proteins, leading to a strain with much lower photoinhibition and greater biomass production [81,82].

### 4.3. Engineering Membrane Transporters for Improved Secretion and Export of Synthesized Biofuels

Once primary biology-based concerns in biofuels production such as inefficiencies arising from protective LHC mechanisms have been addressed, a second and often even more problematic challenge is physically obtaining the product from the host cell. Indeed, one of the most expensive steps in the downstream processing of biofuels is the harvesting and extraction of the product from biofuel-synthesizing cells. Consequently, intense research efforts focus on altering cellular membranes and membrane-bound transporters to facilitate the secretion of biofuels [83]. Unfortunately, although several genes involved in secretory pathways have been identified several years ago, their mechanisms are not well defined [84,85,86,87]. However, the ABC transporters, a family of plant wax transporter, still hold promise as a means of secreting long-chain alkane, ketone, aldehyde, alcohols, and possibly fatty acids from cells due to their promiscuous gating properties [88,89,90,91]. It is hoped that the secretory properties of these proteins can be further tuned by the introduction of site mutations and further, that the engineered transporters can be functionally expressed in species that are good lipid producers to aid in cost effective production of biofuels. Once a secreted biofuel has been successfully exported from a cell, a final, but nevertheless important precaution is to ensure that contaminating organisms capable of using the fuels for a food source are absent from the culture system to avoid lowered yields [83].

### 4.4. Circumventing Biomass Transport for Improved Efficiency of Biofuel Production

Rather than produce biofuels within a cell, which involves facing the challenges associated with the extraction of the synthesized product, another option is to produce the product outside of a cell by having the requisite enzymes secreted from a cell or embedded into the plasma membrane such that their catalytic domains are displayed on the cell surface. This strategy not only reduces the costs involved with extraction of a biofuel from a host cell and subsequent purification from cellular debris, but also bypasses limitations in substrate uptake. Limitations in substrate uptake are especially acute in situations where the cell used in the energy extraction process is not actually the primary organism that harvested the solar energy; an illustration is a corn plant that uses photosynthesis to produce complex lignins, cellulose, hemicellulose and other forms of complex biomasses. A major challenge is that most “raw substrates” such as these are large insoluble materials that cannot be taken up and utilized by the micro-organisms that process them into useable biofuels and the process of converting bulk biomass into nutrients appropriate for microorganism processing represents one of the highest energy costs of the biofuel process [92].

To improve biofuel production from “raw” plant matter, an attractive solution is to engineer cells that display enzymes necessary for the breakdown of complex biomacromolecules on their membranes with the catalytic domains oriented into the extracellular space. Cell surface technologies suitable for this purpose were initially developed for antigen presentation to facilitate vaccine production [93,94], but researchers realized that this strategy could be easily adapted to improve biocatalysis from industrial catalysts to sorbents and sensors [95]. For example, appropriate surface display can be achieved by using the C-terminal half of α-agluttinin, a yeast membrane protein responsible for mating type. Using this technique, strains of *S. cerevisiae* were engineered to co-display glucoamylase and α-amylase, thus allowing the yeast cells to process starch as a substrate [96,97,98]. Using a different strategy, β-glucosidase was displayed on the surface of yeast cells to facilitate conversion of cellubiose into ethanol. It is worth noting that similar approaches have enabled cellulose fermentation through installation of cellulolytic enzymes into the cell membrane of yeast strains [99]. Other yeast-based biocatalysts that express Xylanase II on their surface have been developed and are capable of fermenting xylan into ethanol [100]. Another method to achieve surface display is to fuse proteins onto the flocculation functional domain (Flo1p). This strategy has been used to express glucoamylase and α-amylase on the surfaces of yeast cells allowing direct fermentation of ethanol from raw corn starch [97]. These research efforts are now beginning to allow saccharification (break down of large complex glycans into simple sugars [101]) and fermentation to be carried out in one step and achieve the eventual goal of “consolidated bioprocessing” [95,102].

### 4.5. Biocapacitors and Biofuel Cells through Membrane Engineering

Biofuel cells are energy devices inspired by nature in their use of naturally occurring food sources as fuel but differ from the systems discussed above insofar as their goal is to produce electricity rather than chemicals or liquid fuels. In addition, although “biofuel cells” typically use enzymes in this process, they can also incorporate living, biological cells (usually fungi) into their design; in either case electricity is produced by oxidizing a variety of organic substrates. Because these systems can be designed to consume organic components of waste materials, they hold promise as an attractive way to generate an environmentally friendly power [103]. However, poor electron transport and limitations in mass transport are serious challenges that need to be overcome to develop viable biofuel cells and realize the commercial potential of this emerging technology. Most efforts to tackle these problems have focused on optimizing the characteristics of electrodes [104,105]. However, with the success of microbial approaches in biofuel synthesis, researchers are now starting to employ cell engineering strategies to improve efficiency [106]. While most microbial approaches to date have focused on understanding electrical mechanisms and selecting effective organisms [107,108], there remains an unmet need to create strains that are more highly electroactive by genetic engineering or altering membrane properties to improve electron transfer efficiency. One membrane engineering strategy to improve electron transfer efficiency in biofuels cells is to increase the number of cytochrome complexes, which shuttle electrons across membranes, in the outer membrane [106]. So far, membrane engineering approaches have not been utilized widely in the preparation of biofuel cells but, for biofuel cells to reach their full potential, they will likely need to incorporate strategies such as the surface display of cellulolytic enzymes (as described above) to ameliorate the need for pretreatment of abundant potential feedstocks, such as raw plant materials.

### 4.6. Developing Artificial Nano Assemblies to Convert CO_2_ and Water into Fuel

Artificial photosynthesis attempts to replicate the natural process of photosynthesis that converts sunlight, water, and carbon dioxide into carbohydrates and oxygen [109]. The term broadly refers to any strategy for capturing sunlight and storing the energy in the chemical bonds of a fuel. Not surprisingly, membrane-based approaches play an important role in these efforts; for example, in the organization of nano-assemblies designed to recapitulate photosynthesis using artificial components in place of living organisms. Light can be harvested using synthetic antennas that use porphyrin rings as charge donors, fullerenes as charge acceptors, and quinones as proton shuttles, which can all then coupled to an ATP synthase that uses the charge gradients generated to produce ATP [110,111]. The entire set of components self assembles under suitable conditions within a lipid bilayer thus producing an “artificial membrane based chemical factory” [67]. Like many topics covered in this section, these efforts are in nascent stages and far from commercial reality but nevertheless hold tremendous promise as biology-inspired technologies continue to proliferate.

## 5. Biosensors

Biosensing, in the most rudimentary sense, describes technology that contains a biological component used for the measurement of a biological or chemical signal. Cell-based biosensors offer a rapid, reliable and cheap method to simultaneously test for multiple toxins that pose a threat to human cells. The specificity of cell-based biosensors has been largely dependent on the use of surface receptors on cell membranes to measure secondary responses to ligand molecules [112]. Accordingly, the modulation of cell surface receptor expression through surface engineering or genetic modification to tune the specificity as well as the sensitivity of cell-based biosensors has been critical in advancing this technology. In addition, there is the need for—as well as appropriate methods to convert—a biological signal to an electric or photonic signal (as covered in Section 5.1). In more sophisticated systems, the promise of exquisite and specificity in detecting minute changes in physiological conditions by exploiting a functional network of cells is covered in Section 5.2 and one application of biosensors—environmental monitoring—is covered in Section 5.3. Finally, in Section 5.4 emerging efforts to develop membrane-based microarray technologies for high-throughput biosensing applications are outlined.

### 5.1. Surface Engineering Cells for Bioelectric and Photo Sensing

**Figure 5:**
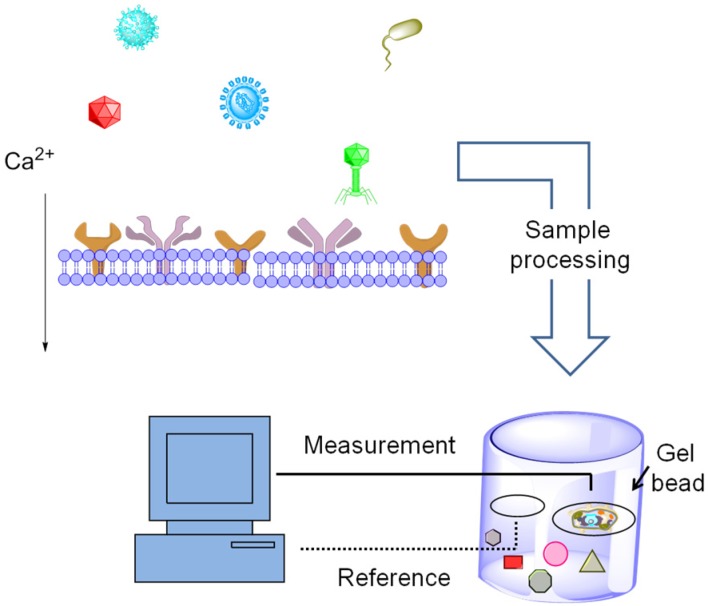
Conceptual illustration of the Bioelectric Recognition Assay (BERA). The first step in developing a BERA is the electroinsertion of antibodies against a specific pathogen (or other entity to be detected) into a biological membrane, often in living cells (top). Once the cells are membrane-engineered with a surface-displayed antibody, a sample can be measured for the presence of a pathogen by encapsulating the engineered cell into a gel bead (bottom, right). Upon ligand binding to the antibodies, the membrane structure is sufficiently altered to change calcium flux and cause a measurable difference in the membrane potential, which can be detected with high sensitivity.

A fundamental requirement for using cells as biosensors is an ability to convert a chemical, biochemical, or biological signal into a “physical” signal such as an electrical signal or a light-based readout. A novel biosensory method was reported in 2001 for detecting chemical and biological molecules by monitoring the electrophysiological interactions of the compounds with immobilized cells. This method was capable of detecting a range of targets including hepatitis C virus in human blood, plant viruses, and the herbicide glyphosate in aqueous solutions rapidly (in 3–5 min) and sensitively (at <100 pg/mL) [113]. This type of electrically transducing cell-based biosensor, known as a bioelectric recognition assay (BERA, illustrated conceptually in Figure 5), utilizes antibodies in mammalian cell membranes to achieve specificity. Insertion of the antibodies can be achieved through electroporation, a process in which an electrical pulse causes an opening of the pores on the cell membrane or osmotic insertion [114] to introduce antibodies onto the surface of a cell resulting in a measurable change in the cell’s membrane potential in response to changes in ligand binding of inserted antibodies [115].

The ability to modify a cell surface with pathogen-specific antibodies allows mammalian cells to act as biosensors for the detection of a variety of viruses and pathogens. In addition to BERA, another biosensor approach that relies on immunoglobulin specificity is the Cellular Analysis and Notification of Antigen Risk and Yields (CANARY) sensor [116]. This method uses B-cells to produce for immunoglobulin M engineered to bind to target pathogens that is also cross-linked with a bioluminescent protein that emits light upon increases in intracellular calcium concentration that occurs upon ligand binding. In another approach, mast cells from mice have been engineered to express receptor proteins specific for *E. coli* [117]; subsequently, fluorescent dye introduced into the mast cells is expelled via cellular exocytosis upon binding with *E. coli*. One use for these biosensors is for the detection of pathogens in food samples [118] such as fried rice and hot dogs. This approach has successfully distinguished active toxins from the inactive ones, and similarly, severe pathogens from their more benign counterparts with detection limits as low as 10^2^–10^4^ colony forming units per gram (CFU/g) and toxins in amounts as low as 10–40 ng.

### 5.2. Exploiting Cellular Networks for Biosensing

The nervous systems of higher animals, which consist of a complex balance of ionic fluxes and electrical impulses transmitted through an intricate network of neuronal cells, have inspired the pursuit of neuronal-based electric biosensors that have extraordinary sensitivity towards external chemical presences. These sensors utilize functional assays that couple the excitable property of neurons with computational genomics as a means to analyze neuronal exposure to environmental toxins [119,120]. Specifically, sensomimes—neurons with engineered cDNA for chimeric receptors with enhanced ligand binding activity—show supersensitivity to foreign molecules [121] including dissolved compounds with such low vapor pressures that they cannot even be detected by dogs. Not only can chimeric mutations increase sensitivity, but they also can be used to examine highly toxic substances because of their fast response time that—coupled with the use of a neuronal network (at least 500 neurons)—ensures that small scale cell death or damage does not affect the overall viability of the biosensor network. Thus, neuronal networks with surface modified chimeric receptors comprise an enticing platform for constructing bioelectric sensors with fast response times and high levels of sensitivity.

For sensomines, or similar cell-based sensing networks, to become robust, easy-to-deploy biosensors, several practical hurdles must be overcome. For example, reproducible and low cost methods to seed neurons onto microelectrode arrays and have them grow into functional neuronal networks are needed. In one strategy O’Shaughnessy *et al.*, formed neuronal networks from neural stem cells that gave rise to multitudes of synaptically interlinked neurons with supporting astrocytes [120]. The network responded positively to GABA_A_ agonists as well as various other inhibitors, thereby indicating the presence of functional synaptic connections. Importantly, these neuronal networks that were grown from neural stem cells functioned similarly to those derived from primary neurons, thus providing a foundation for translation into use for biosensors. Once basic techniques are in place to construct neuronal networks, increased insight into their function is needed; one way to achieve this objective is through *in silico* modeling techniques that combine stochastic ion channel models with whole-network models that predict the bioelectrical response of neuronal networks [122]. Specifically, the model predicted how metabolic networks and electrical activity would respond to external signals and pH changes and thereby provide insight into how living cells can distinguish between a target signal and non-specific stimuli in a complex physiological milieu.

### 5.3. Surface Engineered Microbes as an in Vitro Tool for Environmental Monitoring

Environmental monitoring, which is becoming an increasingly important economic sector, requires quick and precise tools. For example, the detection of toxins and pathogens in food products as well as in water supplies is an urgent—and in many cases still unmet—public health concern. Traditional methods of pathogen and toxin detection and identification for these purposes rely on low throughput and cumbersome techniques such as the polymerized chain reaction (PCR) and culture techniques that often require counting colonies. Environmental monitoring has also been carried out by plant and animal-based assays utilizing algae, plants, as well as fish. However, many times these assays can be expensive and time consuming making them implausible to run on sufficiently large scales often needed for consistent and widespread environmental monitoring. In general, it is crucial that detection assays are sensitive to even the smallest concentrations of harmful compounds or pollutants, can be completed rapidly, and are cost-effective. The shortcomings of current efforts to detect pathogens, toxins, and pollutants in these regards have spurred increasing efforts towards using biosensing technology for higher throughput, simplicity of detection, and fast response times. Bioluminescent bacteria used as biosensors have favorable speed and cost considerations and also compare favorably to more traditional assays that employ algae and fish [123]. Similarly, stress-responsive bacterial biosensor arrays are being developed for the nanoscale detection of copper [124], a metal that is a necessary plant nutrient in small amounts but toxic in elevated amounts. Accordingly, it is important to have a quick and accessible assay to accurately measure copper availability in soil.

An attractive means of monitoring environmental pollutants, especially fresh water streams, is through the measurement of the five day biochemical oxygen demand (BOD). BOD sensors were first developed over 40 years ago but the sensors themselves remain unstable and the measurements are often difficult to reproduce [125]. The sensors have three main parts: the detector, a transducer, and the catalyst. The catalyst used is often a microbe, another type of living cell, or even an isolated enzyme. A drawback of using microbes as the catalyst component is a limited shelf-life, thus the support material used for immobilization plays a crucial role in the commercial feasibility of these biosensors [126]. A comparison of membranes prepared by immobilizing bacteria in polyvinyl alcohol (PVA) cross-linked with glutaraldehyde or immobilized with nitrocellulose or agarose followed by deposition on nylon membranes showed that stability and viability—qualities correlated with its subsequent shelf-life—were maintained up to 400 days, a timespan that will facilitate the commercialization of BOD biosensors. In addition to shelf life, response times of BOD biosensors are an important design consideration. Microbial BOD biosensors have response times of three to five minutes, a substantial improvement from the five days required in early BOD tests [127]; traditional BOD testing was slow because it required the passive diffusion of oxygen into samples. To overcome this limitation, micro-cellular polymer disks with microorganisms seeded onto the micropores were developed wherein the growth of a membrane-like layer of microorganism increased overall surface area while reducing the diffusion path of incoming molecules, thereby increasing microreactor efficiency [128]. BOD biosensors illustrate just one of many efforts to create efficient biosensors that maximize sensitivity, minimize detection time, and have a high degree of stability that enables the long shelf life required for commercialization. 

### 5.4. Towards Cell-Free, High-Throughput Membrane-Based Biosensors

Similar to many of the other emerging membrane engineering applications outlined in this article, cell-free strategies have distinct advantages over cell-based systems for biosensor development. In particular, conversion of this technology to a microarray format promises unprecedented opportunities for rapid and high-throughput detection and sensing platforms. In the past few years, several efforts have been reported towards this goal. In one example, supported lipid bilayers (SLBs) have been deployed in microarray format by first converting the SLBs into spherical supported lipid bilayers (SSLBs) on submicron-sized silicon dioxide beads [129]. Beads can then be deposited individually into an array of micro-fabricated submicron-diameter wells that are just large enough to accommodate a single bead using a “squeegee” technique to clean the substrate surface, while leaving behind SSLBs that have settled into microwells [130]. The value of the SSLB microarray has been demonstrated for characterizing the interaction of cholera toxin with ganglioside GM1, which is a glycolipid easily incorporated into SLBs. In the future, it is anticipated that SSLB microarrays will incorporate membranes that include additional biological macromolecules including membrane proteins and will be particularly attractive for sensing applications and mimicking cell-cell interactions [129,130].

The development of automated microfluidic methods to create planar membrane assemblages with embedded proteins [131] is opening the door to a second basic type of membrane-based microarrays based on the formation of a nanopore that can be exploited for biosensing applications. Several iterations of this approach have been reported since the concept was demonstrated using microarray systems that enabled sub-picoliter, simultaneous monitoring of multiple ionic currents through transmembrane a-hemolysin nanopores arrayed in bilayer lipid membranes [132,133]. For example, formation of lipid bilayers inside microfluidic channel arrays for monitoring membrane-embedded nanopores of phi29 DNA packaging nanomotor has been reported [134] and recent efforts now enable parallel and high-resolution single-molecule detection by single nanopores in suspended molecular membranes densely arrayed in formats that allow high-resolution electrical recording [135]. Intriguing variations of this technology, for example single-molecule mass spectrometry-based detection of different-sized poly(ethylene glycol) (PEG) molecules, provide this technique with promising potential for many elegant and acquisitively sensitive biosensing applications in the future.

## 6. Engineered Membranes for Use in and the Development of Bio 3D Printing Applications

Recent advances in three dimensional printing techniques have made it possible to design increasing complex biomaterials for use in cell and tissue engineering. Three dimensional printing began as a way to manufacture simple 3-D geometrical shapes but is now capable of implementing complex and elaborate network designs. Bio 3-D printing refers to the use of this technology to produce biological constructs ranging from bones and artificial skin to biomimetic membranes, which is of much interest to the field of cell and tissue engineering because this methods provides a new frontier for the development of tissue and customized cell membranes. Bio 3-D printing allows the creation of three-dimensional geometric structures made from aqueous solution droplets joined together by single lipid bilayers such as liposomes, which are vesicle-like droplets surrounded by a lipid monolayer approximately one picoliter in size. The liposomes are printed in layers on top adjoining their monolayers to form a lipid bilayer between them. Their lipid bilayers can be functionalized into highly engineered 3-D scaffolds by adding membrane proteins, and establishing electrical communication across the membranes. Networks can consist of tens of thousands of liposomes with at least several thousand layers, thus forming a tissue-like printed material.

A basic 3-D bio printer consists of two glass capillary nozzles with thin needle tips with each joined to a chamber filled with an aqueous solution and capable of providing droplets of different aqueous compositions. Computer-controlled electronics modulate voltage to a piezoelectric transducer located in the back of the nozzles to administer the droplets [136]. The printing takes places in oil because oil allows the aqueous droplets coated with lipid monolayers to adhere to each other and form stable lipid bilayers. Printing can either be done in bulk amounts of oil or in oil droplets depending on the desired geometry of the final structure. For example, if a cuboidal figure is desired, the network would have to be built in horizontal layers. In order to achieve such a pattern, horizontal layers of droplets can be printed in a horizontal printing surface submerged in a bath of lipid-containing oil [136] whereas a more complicated shape such as a sphere can created by printing inside an oil drop suspended in an aqueous solution. The oil drop is kept in place by placing a frame loop around the drop [136,137]. The excess oil can then be removed by suction through one of the printing nozzles once the printing process is complete.

If a tissue is to survive and adapt to its environment, the cells in the tissue need to have open communication with the environment and be able to transport molecules through their membrane compartments when necessary. Exchange of materials across the membrane and electrical communication are very important for the functionality of biomimetic membranes. Printing stable networks that mimic real tissue functions can be achieved through the insertion of membrane proteins in printed lipid bilayers and osmotic water flows can be manipulated by varying salt concentrations. Membrane conductivity has been achieved through the insertion of wild-type Staphylococcal α-haemolysin (αHL) into the droplets of aqueous solution [136]; αHL is a transmembrane transport channel consisting of an anti-parallel β-barrel that can be used to transfer small molecules across the bilayers of lipid vesicles [136,137,138]. The αHL pore can also create an ionic conductive circuit across otherwise insulating bilayers in droplet networks allowing compartments to communicate with each other and the entire assembly to communicate with the external environment. If electrodes are placed on either side of the αHL circuit, they are able to conduct a current through the transport channels [136,137]. The current varies with pH and salt concentrations and also by applying different voltages through the electrodes. The addition of an αHL circuit allows electrical communication to take place between a droplet and its surrounding solution, or between two droplets in a network. Using these membrane proteins along with 3-D printing technology, an electric circuit can be printed with definite circuit paths, which is analogous to a neuronal circuit.

Osmosis is the movement of water from a region of low solute concentration (high water concentration) to a region of high solute concentration (low water concentration) and osmotic balance must be maintained to print a stable and useful biomimetic membrane. Living cells use several different methods to avoid swelling due to osmosis, for example they contain water pores and channels that regulate osmotic balance and thus prevent swelling or shriveling. Biomimetic membranes also have to be able to maintain osmotic balance and once again, transmembrane proteins are used to control osmotic flows by transporting solute in and out of the cell, and thus, maintain osmotic balance.

Osmosis can also be manipulated as an important tool in engineering droplet networks to attain certain geometries that can be difficult to print directly. The lipid layer of each droplet surrounds a certain amount of aqueous solution which can be tailored to specific printing needs. A higher concentration of salt in one droplet than in the surrounding droplets will cause it to swell with water while the surrounding cells shrink accordingly until the salt concentration is the same for all droplets in the network. The osmotic flows of water across the bilayers of the droplets will alter the conformation of the network through self-folding. For example, a planar flower shaped membrane with four petals can be printed in which the lower layers have lower osmotic gradients than the upper layers. This will cause the lower droplets to swell causing the network to fold inward and closing the flower creating a stable and self-supporting hollow sphere.

If osmotic flows could made reversible and controllable, a network of droplets could be printed that would fold and contract similar to other tissues in the body. A way to pursue this objective is through printed biomimetic membranes designed to display droplet sensitivity to pH and temperature changes. A step in this direction is by engineering the lipid bilayers of the droplets to release their contents at a certain pH [137]. This function is very promising for the selective release of drugs or other chemicals inside the body due to a direct stimulus. A pH-sensitive lipid bilayer can be constructed from a lipid that favors a non-bilayer state, such as 1,2-dioleoyl-*sn*-glycero-3-phosphoethanolamine (DOPE), and a lipid that stabilized the bilayer at a pH above its pK_a_, such as oleic acid [137]. Such a bilayer remains stable provided that the pH of the surrounding solution is greater than the pK_a_ of the oleic acid but destabilizes at lower pH due to the protonation of the oleic acid. When the pH becomes lower than the pK_a_ the lipid bilayers break apart, releasing the internal droplet contents and mixing them with the external solution. The release of the contents across networks of droplets can be synchronized, with the simultaneous release of contents occurring within a minute [137]. Droplet rupture can also be triggered by temperature with the temperature that determines the breaking of the membrane and release of its content tuned by the composition of lipid forming the bilayer. Droplets can be synchronized to burst within 0.1 degree Celsius of each other, making temperature an efficient and localized release mechanism. Both pH and temperature-triggered releases can be achieved without fusion of the printed droplets, keeping their compartments closed to each other and avoiding the mixing of contents [137]. Finally, hollow membranes with these properties could be printed to deliver prodrugs that need to be present in the body to produce a drug but are too unstable to be administered through intravenous drip or pill form.

There are many complicated factors affecting the function and conformation of networks of lipid droplets. Fortunately, predicting the final geometrical shape of the droplet network is possible through the use of computational models. Three-dimensional printer software is now able to determine the final geometry fairly accurately by taking into account the initial geometry, distribution of two types of droplets, and osmotic gradients. Through cell membrane engineering droplet networks have been given electrical communication by transmembrane proteins, such as αHL, and macroscopic folding due to osmotic flows. These properties are essential for manipulating biomimetic membranes and making them similar to real cells and tissue and increasingly, once a highly engineered membrane can be designed, three-dimensional printing can create it.

## 7. Summary and Conclusions

One of the most important cellular architectures is the plasma membrane, which not houses an entire cell, but also provides a unique environment for transmembrane proteins. Although bioscience is becoming increasingly proficient at manipulating genomes and systems for protein production in many types of cells and in cell-free expression system, serious challenges remain for membrane proteins. For example, as discussed in Section 3, these proteins require their native or native-like environments for proper folding and function motivating efforts to engineer cellular systems to increase bilayer biogenesis in protein overexpression systems. While membrane engineering in living cells is experiencing exciting advances, inherent problems exist, such as waiting times needed to grow cells, short shelf lives of many products, and the need for sterility that are spurring complementary cell-free engineering approaches. For example, polymersomes and NLPs are achieving success as framework substitutes for the cellular membrane in transmembrane protein production systems. Avoiding the use of cells offers potential for reductions in cost, increased throughput, and increased control of the microenvironment during assays based on membrane proteins.

Increasing success in these efforts is expected to pay off in a range of applications from drug development to biofuel production strategies (Section 4). Insertion of engineered proteins into the surface membranes of cell-based systems holds great promise for energy production. For example, in conventional cell-based biofuels platforms where intracellular metabolism is engineered to produce biofuels, concomitantly engineering transporters into cellular membranes to extract the biofuel increases efficiency. In a more sophisticated approach, display of biomass-processing enzymes useful in the conversion of biomatter into biofuels on cell surface membranes can circumvent these transport problems all together. In a different direction, membrane technologies are critical for advancing cell-based biosensors (Section 5) that can be fabricated for toxin screening, bioelectric and photo sensing, and environmental remediation purposes by inserting even more radically different proteins into the surface of cells.

Despite the diversity of applications covered in this review, a common theme is the use of biological—or biology-inspired artificial—membranes in the design and assembly of *in vitro* tools that in some cases utilize living cells and in other cases are completely synthetic. The new and exciting field of bio 3-D printing (Section 6) essentially combines many aspects of the previous discussed topics, including a novel way to insert membrane-bound proteins into synthetic lipid bilayers. Through ingenious design and approaches, bio 3-D printers are now capable of printing complex shapes and sheets of material that behave in a tissue-like manner. Bio 3-D printing as an *in vitro* tool stands to revolutionize our understanding of biology by allowing one to specifically manipulate cell mimicking structures in not only a 3-D environment, but also within a complex tissue-like environment, thus opening the door for advanced investigation of—and control over—cell-cell communication and behavior in various tissue architectures and environments.

In the future, technology may advance to the point we will be able to construct functional cells through entirely synthetic means. Achieving this tremendous accomplishment will require additional advances in technology but also continued investigation of fundamental concepts, such as the fact that the cell membrane is one of the most important features of a cell that controls signaling cascades, temporal cues, cell-cell communication, cellular adhesion, and cell motility. At the present time, *in vitro* tools and reagents based on engineered membrane systems already play important roles in the design, understanding, and discovery of medicines, biofuels production, and environmental monitoring, as outlined in this article.
